# Development of macular retinoschisis long after the onset of retinal arterial occlusion (RAO): a retrospective study

**DOI:** 10.1186/s12886-018-0730-5

**Published:** 2018-02-27

**Authors:** Norihiko Ishizaki, Teruyo Kida, Masanori Fukumoto, Takaki Sato, Hidehiro Oku, Tsunehiko Ikeda

**Affiliations:** 1 Department of Ophthalmology, Yao Tokushukai General Hospital, 1-17 Wakakusa-cho, Yao, Japan; 2 Department of Ophthalmology, Osaka Medical College, 2-7 Daigaku-machi, Takatsuki, Osaka, 569-8686 Japan

**Keywords:** Retinal artery occlusion (RAO), Optical coherence tomography (OCT), Macula, Retinoschisis

## Abstract

**Background:**

To describe a retrospective study of macular retinoschisis that developed long after the onset of retinal artery occlusion (RAO) using optical coherence tomography (OCT).

**Methods:**

We describe changes in macular findings and visual acuity (VA) of 29 patients (21 males and 8 females, mean age: 66.1 ± 16.9 years) with RAO (18 branch RAOs [BRAOs] and 11 central RAOs [CRAOs] who visited Osaka Medical College Hospital over an 8-year period based on a medical chart review.

**Results:**

The mean VA (logMAR) increased from 1.06 ± 1.08 (CRAO: 2.04 ± 0.99; BRAO: 0.37 ± 0.40) at the first visit to 0.71 ± 0.87 (CRAO: 1.46 ± 0.86; BRAO: 0.18 ± 0.30) at the final visit. Macular OCT revealed swelling or hyper-reflectivity of the inner retina in the early phase of RAO and retinal thinning in the late phase. Among the 29 patients, two patients (a patient with BRAO and a patient with CRAO) developed macular retinoschisis about 1 year after RAO onset. The VA of the patient with BRAO was 20/300 at the first visit, and it improved to 20/25 two days after onset following eye massage and anterior chamber paracentesis. However, his VA worsened, declining from 20/25 to 20/50, and retinoschisis occurred 13 months after RAO onset. The patient with CRAO showed macular changes including small cystoids at the first follow-up visit more than 3 weeks after onset and developed retinoschisis 11 months after the first visit. In addition, two patients with BRAO and one patient with CRAO developed macular changes including small cystoids 3 weeks after onset, with the BRAO complicated by retinal vein occlusion. In the CRAO patient, the cystoid macular edema was resolved 1 month after the first visit.

**Conclusions:**

Macular retinoschisis is unusual, but a possible complication of RAO that can develop long after the onset of the occlusion, potentially resulting in renewed VA deterioration.

## Background

Retinal artery occlusion (RAO) is induced by emboli and usually occurs suddenly. Systemic diseases, such as systemic hypertension, myocardial infarction, carotid artery stenosis, and diabetes mellitus, can be risk factors for RAO [1–4]. The diagnosis of RAO is based on sudden visual loss and characteristic fundus findings [5]. The ophthalmoscopic findings of acute-phase and late-phase RAO have been described in detail elsewhere [6].

Optical coherence tomography (OCT), which is a noninvasive technology, can be used to produce in vivo cross-sectional images of the retinal microstructure [7]. Macular OCT findings are helpful for determining the degree of retinal damage and predicting the prognosis of visual acuity (VA) in early- and late-stage RAO. A previous study reported that macular OCT showed swelling or hyper-reflectivity of the inner retina in the early phase of RAO, followed by retinal thinning in the late stage [8]. Such macular OCT findings are seen in most RAO cases.

In this retrospective study, we describe macular retinoschisis and macular changes including small cystoids detected by OCT long after the onset of RAO.

## Methods

This retrospective study was approved by the Institutional Review Board (IRB) (the Ethics Committee of the Osaka Medical College (No. 2034)), and the tenets of the Declaration of Helsinki were followed.

For this retrospective study, we reviewed our medical charts of RAO patients who visited Osaka Medical College hospital from April 2008 to May 2016. In the initial examination, each patient underwent a comprehensive ophthalmic examination that included the measurement of their best-corrected visual acuity (VA) using a Landolt chart and retinal findings using fundus biomicroscopy with a non-contact lens. In each follow-up visit, patients underwent a comprehensive ophthalmologic examination that included the measurement of their best-corrected VA, color fundus photography, and macular OCT examination; an additional fluorescein angiography (FA) was performed if deemed necessary.

## Results

Changes in macular findings and visual acuity (VA) of 29 patients (21 males and 8 females; mean age: 66.1 ± 16.9 years) with RAO (18 branch RAOs [BRAOs] and 11 central RAOs [CRAOs]) were investigated in this study. Systemic complications were as follows: systemic hypertension (*n* = 17, 54.8%), myocardial infarction (*n* = 7, 22.6%), diabetes mellitus (*n* = 5, 16.1%), stenosis of the carotid artery (*n* = 5, 16.1%), cerebral infarction (*n* = 4, 12.9%), postsurgery heart valve replacement (*n* = 4, 12.9%), atrial fibrillation (*n* = 2, 6.5%), atherosclerotic obstruction (*n* = 2, 6.5%), postsurgery synthetic blood vessel graft in the aorta (*n* = 1, 3.3%), and anemia (*n* = 1, 3.3%). Macular findings were assessed by optical coherence tomography (OCT) (Spectralis, Heidelberg, Germany). The mean VA (logMAR) improved from 1.06 ± 1.08 (2.04 ± 0.99 for CRAO and 0.37 ± 0.40 for BRAO) at the first visit to 0.71 ± 0.87 (1.46 ± 0.86 for CRAO and 0.18 ± 0.30 for BRAO) at the final visit. The macular OCT findings showed swelling or hyper-reflectivity of the inner retina in the early phase of RAO, followed by thinning of retina in the late phase.

**Fig. 1:**
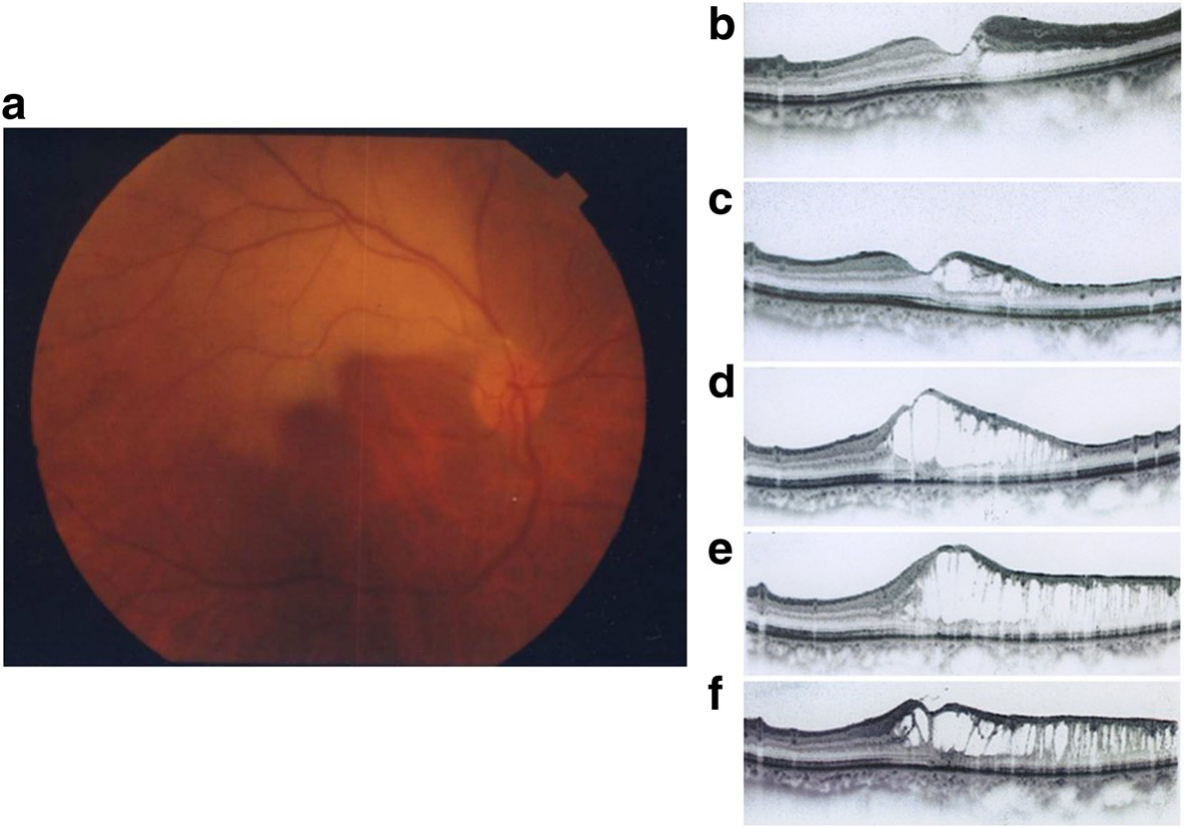
A case of BRAO with retinoschisis. **a** Fundus photograph at the first visit. **b** Macular OCT image 1 month after the patient’s first visit. The patient’s VA was 20/25, and hyper-reflectivity of the inner retina was observed. The outer nuclear layer and Henle’s layer were detached. **c** 9 months: Retinoschisis and thinning of the inner retina were observed. The VA was 20/25. **d** 13 months: Retinoschisis had spread and involved the fovea. The VA had dropped to 20/40. **e** 15 months: Retinoschisis was widespread. The VA was 20/40. **f** 16 months: The VA was unchanged

Of the 29 patients, two patients (a patient with BRAO and a patient with CRAO) developed macular retinoschisis about 1 year after RAO onset. In the patient with BRAO, his VA was 20/300 at the first visit, and it improved to 20/25 two days after the onset, following eye massage and anterior chamber paracentesis performed at the first visit. Figure 1 shows the macular changes revealed by fundus photography and macular OCT at the first visit. However, the patient’s VA worsened, declining from 20/25 to 20/50, and retinoschisis recurred 13 months after onset. The retinoschisis remained 16 months after the first visit.

**Fig. 2:**
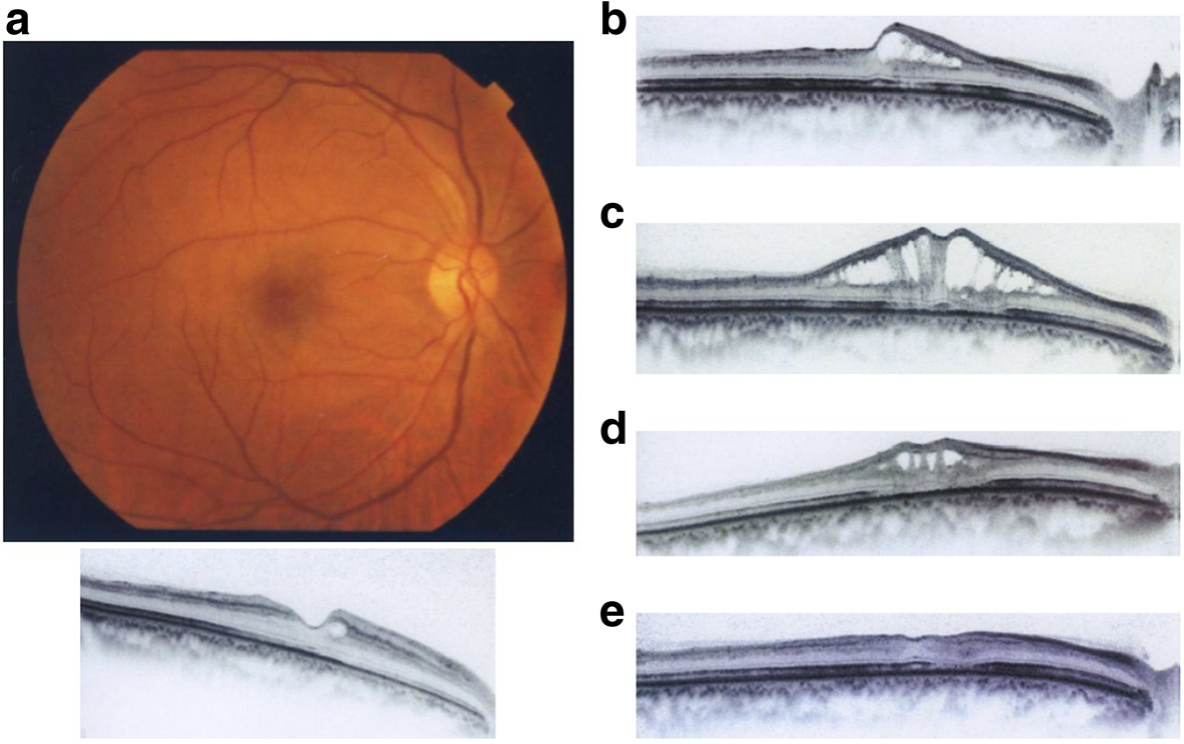
A case of CRAO, with macular change including a small cystoid. **a** Fundus photograph and macular OCT image at the first visit. The VA was 30 cm according to the finger-counting method. A small cystoid was observed in macular OCT 3 weeks after onset. **b** 11 months: Retinoschisis developed following the macular change including a small cystoid. The VA was 20/1000. **c** 18 months: Retinoschisis had spread. The VA was unchanged. **d** 19 months: Retinoschisis had decreased. The VA was unchanged (20/1000). **e** 22 months: Retinoschisis was resolved, but the VA was not improved. This figure was previously published in *Ganka Rinsho Kiyo* [18]. Ganka Rinsho Kiyo-kai granted permission

In the patient with CRAO (Fig. 2), macular change including a small cyst was observed at the first follow-up visit 3 weeks after onset, and his VA was remained 30 cm according to the finger counting test. Eleven months after the first visit, the patient developed retinoschisis, and his VA was 20/1000. The retinoschisis disappeared 22 months after the first visit, but the patient’s VA did not improve.

**Fig. 3:**
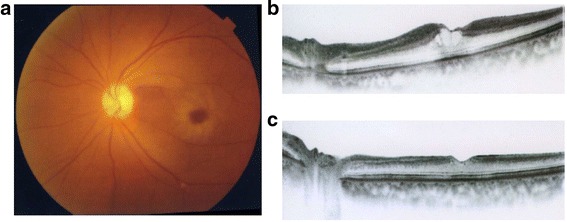
A case of CRAO with cystoid macular edema. **a** Fundus photograph at the first visit. **b** Macular OCT image at the first visit. At least more than 3 weeks had passed from the onset. Cystoid macular edema was observed, and the VA was 20/300. **c** 1 month: The cystoid macular edema had disappeared, but the VA had dropped to 20/600

In addition, two patients with BRAO and one patient with CRAO showed cystoid macular changes 3 weeks after onset. Two of these BRAOs were complicated by retinal vein occlusion. In the CRAO patient, cystoid macular edema was observed at the first visit (Fig. 3). The patient visited our outpatient clinic for a second opinion. His VA was 20/300. The cystoid macular edema resolved 1 month after the first visit. However, his VA had deteriorated (20/300 at the first visit and 20/600 at the final visit). We have obtained consent to publish from the three patients described above.

## Discussion

Herein, we described the development of macular retinoschisis in patients long after the onset of RAO. The findings in this retrospective study indicate that some kind of macular changes may occur in some patients with RAO, even after retinal thinning has occurred in late-stage RAO. They also indicate that VA may deteriorate in BRAO patients, despite an initial improvement, due to the occurrence of macular retinoschisis. Clinicians should be aware of the various presentations of macular changes on OCT findings during the acute and late stages of RAO.

There are rare, but a few case reports of paracentral acute middle maculopathy (PAMM) due to CRAO [9]. PAMM is the term recently used to describe the spectral-domain OCT finding of band-like hyper-reflectivity at the level of the inner nuclear layer associated with retinal vascular diseases. In this retrospective study, we had no case of only PAMM, and macular OCT findings in all of our cases showed hyper-reflectivity and/or swelling of the inner retina in the early phase of RAO, followed by thinning of retina in the late phase. Some cases of PAMM might be included; however, the details are unknown because we could not analyze layer-by-layer findings of macular OCT from our every case from 2008 to 2016. This is a limitation of this retrospective study.

There have been a few reports of macular findings of retinoschisis in the late phase of RAO. Pathologically, retinal changes were observed soon after occlusion of the central retinal artery, in addition to necrosis of the nerve fibers, ganglion cells, inner nuclear layer, and inner plexiform layer after a short period of anoxia [10]. In experimental occlusion of the central retinal artery using rhesus monkeys, occlusion for up to 90 min did not result in significant permanent neural damage [11]. However, occlusion of the central retinal artery for 105 min or longer produced irreversible permanent neural damage. Research also showed that neuroglia can be damaged after RAO and that necrotic tissue may be ingested by macrophages and moved through the outer retina, choroid, or recanalized retinal vessels [12]. Müller cells are the primary glial cells of the retina. They extend longitudinally through the retina from the outer nuclear layer to the border of the retina and vitreous [13]. Experimental embolization of the central retinal artery in the owl monkey led to various types of intraretinal schisis [14, 15]. These findings suggest that the formation of retinoschisis in patients with late-stage RAO may be the result of damage to Müller cells and nerve fibers.

In the present study, macular changes including small cystoids were detected in subacute-stage RAO. Such macular findings are uncommon but have been described in earlier case reports [16, 17]. Cystoid macular edema, which is extracellular arising between the inner nuclear and outer plexiform layer, resulting in a “flower petal” appearance, can be seen in acute phase of RAO. Ng WY et al. described that the outer retinal layer is involved as well in CRAO [16]. The pathomechanism is unclear; however, the occurrence of cystoid macular edema means that CRAO does not only affect the inner retina but the outer retinal layer and outer blood-retinal barrier. In the present study, two patients with late-stage RAOs developed retinoschisis following macular changes including small cystoids. In both patients, these changes occurred within 1 month after onset. These changes may be the result of necrosis in the inner retina. All the patients in this retrospective study showed thinning of the retina in late-stage RAO. OCT findings of retinoschisis after RAO are relatively rare. Clinicians should be aware of retinoschisis in cases of late-stage RAO, as it may result in deterioration of VA.

## Conclusion

We described a retrospective study of macular retinoschisis that developed long after the onset of retinal artery occlusion (RAO). Macular retinoschisis is unusual, but a possible complication of RAO that can develop long after the onset of the occlusion, potentially resulting in renewed VA deterioration.

## Abbreviations

BRAO: Branch retinal artery occlusion
CRAO: Central retinal artery occlusion
FA: Fluorescein angiography
OCT: Optical coherence tomography
PAMM: Paracentral acute middle maculopathy
RAO: Retinal artery occlusion
VA: Visual acuity
